# A link between the destruction of the mantle root beneath the North China Craton and the Cretaceous Greenhouse

**DOI:** 10.1093/nsr/nwac049

**Published:** 2022-06-23

**Authors:** D Graham Pearson

**Affiliations:** Department of Earth and Atmospheric Sciences, University of Alberta, Canada

The carbon dioxide level in Earth's atmosphere has long been recognized as a key driver of modern climate. However, the amount of carbon trapped in the solid Earth constitutes over 99.99% of all Earth's carbon and thus dwarfs that in the atmosphere. Recognition of the key role played by this vast source of CO_2_ has increased our need to understand the interplay between the solid Earth and the atmosphere [1].

Cratons are the oldest features of Earth's dynamic surface, containing the oldest exposed rocks, underpinned by strong lithospheric keels at least 150 km thick [2]. This thick lithosphere, stable for >Gyr time periods, is a long-term sink for many elements, including carbon, that can be subsequently reactivated if the cratonic lithosphere is destroyed via redox processes [3].

**Figure 1.**
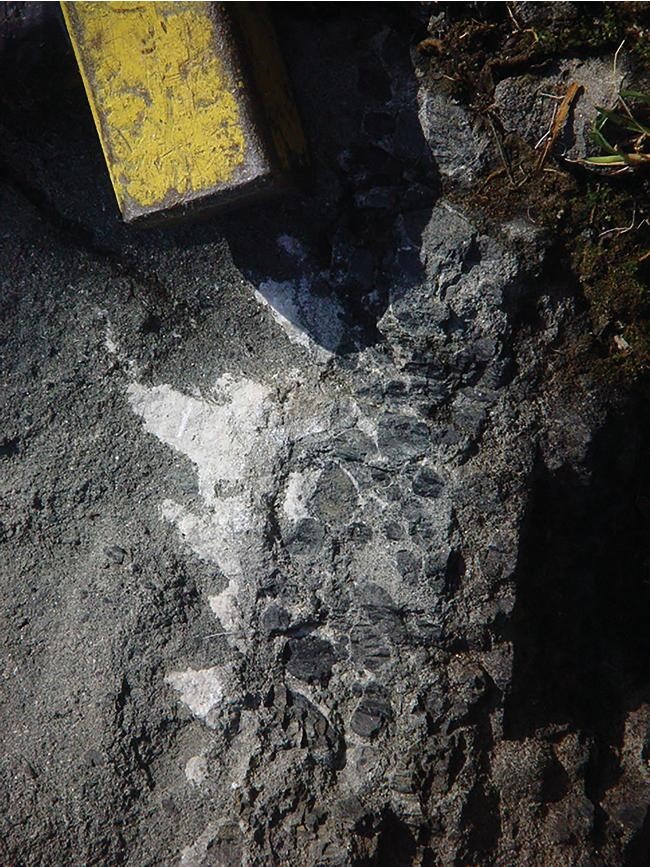
Carbonate-rich ultramafic lamprophyre from southwest Greenland. White specks are carbonate. Field of view: 25 cm.

Cratons sometimes lose their stability through modification of their mantle roots. One of the most striking examples of a ‘modified craton’—in the terminology of Pearson *et al*. [2]—is the North China Craton, or NCC, which lost its deep mantle lithospheric root, in the eastern portion of the craton, in Phanerozoic times [4]. In a novel study, Wang *et al*. [5] assemble a compelling and diverse array of evidence for the major release of CO_2_ during the destruction of much of the lithosphere beneath the NCC. First, they establish that the ancient, thick, pre-Cretaceous lithosphere beneath the eastern portion of the NCC must have contained a significant amount of carbon, from eons of metasomatic enrichment, culminating in the multiple subduction episodes witnessed by this craton in the Phanerozoic. These events led to enrichment of the lithosphere with subducted carbonates, as revealed by magnesium isotopes—a now well-established tracer of recycled ocean sediment carbonate—in the volcanic lamprophyres produced through melting this carbonate. Some lamprophyres, such as the ultramafic lamprophyre group, contain obvious carbon through the carbonate they contain (Fig. 1). Through spectroscopic analysis of gas bubbles within tiny melt inclusions in NCC lamprophyres, Wang *et al*. [5] show that these rocks contained up to 2 wt% CO_2_. Typical basalts have only a few hundred ppm CO_2_. Therefore, the NCC lamprophyres, produced through lithospheric melting, are very CO_2_ rich. Wang *et al*. [5] show that the timing of the eruption of these magmas along with associated crustal heating would have resulted in enormous volumes of CO_2_ released into the early Cretaceous atmosphere, likely accentuating the ‘Cretaceous Greenhouse’ episode.

This remarkable result [5] leads to obvious interest in whether the lithospheric root destruction events associated with numerous other modified cratons, for example those shown in ref. [2], may have also made contributions to ancient spikes in atmospheric CO_2_, and whether these events might be detected in the geological and geochemical records that we are increasingly able to read at high fidelity. The study by Wang *et al*. [5] will act as the trigger for future work.

***Conflict of interest statement*.** None declared.
